# Collodion in Chilblains

**Published:** 1849-08

**Authors:** 


*Collodion in Chilblains.*—In chilblains I have used it with the most decided success. In one case the patient had her feet for some time exposed to severe cold, which produced an erythematous inflammation of several of the small toes. They were swollen, red, tender, and itching. I completely enveloped them in a thick coating of collodion, and the contraction which took place on its drying produced so much compression of the vessels, that the toes were as pallid as frozen ones. The pain and itching were immediately relieved, and in twenty-four hours these members were nearly well. I have cured pernio of the heel, also, with this article, but I do not believe it a panacea for all cases of chilblain, even in its erythematous stage.—Dr. C. Green, in *Buffalo Medical Journal.*

